# Revised environmental identity scale: Adaptation and preliminary examination on a sample of Italian pet owners

**DOI:** 10.3389/fpsyg.2022.892841

**Published:** 2023-01-30

**Authors:** S. Ariccio, O. Mosca

**Affiliations:** ^1^Department of Psychology of Developmental and Socialization Processes, Sapienza University of Rome, Rome, Italy; ^2^Department of Education, Psychology, Philosophy, University of Cagliari, Cagliari, Italy

**Keywords:** environmental identity, Italy, translation, adaptation, pet owners, EFA, Revised EID, scale

## Abstract

The Revised Environmental Identity (EID) Scale is a tool proposed by Clayton in 2021 to replace her 2003’s EID Scale and aims to measure individual differences in a stable sense of interdependence and connectedness with nature. Since an Italian version of this scale was still missing, the present study presents an adaptation of the Revised EID Scale in Italian. The scale has been translated, back-translated, and administered online to 163 pet owners living in Italy in the context of a study about pet attachment. A parallel analysis suggested the existence of two factors. The exploratory factor analysis (EFA) identified the same number of factors: “Connectedness to nature” (nine items) and “Protection of nature” (five items); the two subscales were found to be consistent. This structure explains more variance compared with the traditional one-factor solution. Sociodemographic variables do not seem to affect the scores of the two EID factors. This adaptation and preliminary validation of the EID scale have relevant implications for studies in the Italian context as well as on specific population groups such as pet owners, and more generally, for international studies on EID.

## Introduction

### Environmental identity and its relationship with similar constructs

As a facet of self-identity, environmental identity (EID) is considered an aspect of one’s identity describing individuals’ identification with the physical and natural world, and it is thus viewed as a sub-identity similar to other sub-levels, such as personal identity, social identity, and place identity (Proshansky, 1978; Proshansky and Fabian, 1987; Twigger-Ross et al., 2003). More specifically, EID is defined as “a sense of connection to some part of the non-human natural environment (…) that affects the way in which we perceive and act toward the world; a belief that the environment is important to us and an important part of who we are” (Clayton, 2003 p. 45–46).

Similar to an identity, EID is also defined both as a product and a force (Rosenberg, 1981; Clayton, 2003). It is considered a product because it is the result of personal history and the emotional connection the individual has developed toward the environment. As for the role of EID as a force, Clayton’s and several consequent studies highlight how EID is associated with environmental concern (Clayton and Kilinç, 2013; Clayton et al., 2019) and pro-environmental attitudes and behaviors (Clayton, 2003; Tam, 2013; Dresner et al., 2015; Scopelliti et al., 2018; Clayton et al., 2021). Identifying with nature also implies the perception of similarities between the self and nature, and a sense of connection or emotional attachment to it (Clayton and Myers, 2009; Schultz, 2001). This connection is possibly explained by the biophilia hypothesis, which asserts that the human dependence on nature “extends far beyond the simple issues of material and physical sustenance to encompass as well the human craving for aesthetic, intellectual, cognitive, and even spiritual meaning and satisfaction” (Kellert, 1993, p. 21), and therefore, the self-knowledge the environment provides, satisfies basic psychological needs (Clayton, 2003). Identification with nature is also relevant to the pets as ambassadors hypothesis, initially proposed by Serpell and Paul (1994), which states that owning pets during childhood may lead to positive attitudes toward animals in general, later in life, and to related behaviors, such as becoming members of animal welfare charities, and sometimes, by spill-over effects, even environmental and conservation organizations (Serpell and Paul, 1994; Miura et al., 2002; Auger and Amiot, 2017; Possidónio et al., 2021). Concretely, our connection with nature is in part built on our interactions with animals and the natural world (Clayton, 2003), and animals, in this view, can be considered as a bridge to the consideration and care of the natural world. Contact with animals, both domestic and wild, can promote contact with nature through everyday activities, for example, walking one’s dog or observing wild animals in green areas, urban and/or rural areas (Podberscek et al., 2000).

Different studies have investigated the links between the relationship individuals have with animals and the one they have with nature. For example, Nisbet et al. (2009) reported that feeling to be close to nature is associated with a greater love of animals, and Gosling and Williams (2010) confirmed that assigning greater value to animals is associated with the feelings of an emotional connection with nature. These results are coherent with animals’ conceptualizations as an inherent part of nature (Kellert, 1993; Schultz, 2000; Clayton, 2003).

Auger and Amiot (2017), following and extending the pets as ambassadors hypothesis, proposed that the same mechanisms through which pets act as ambassadors for animals should also apply between animals and nature. It should be noted that the relationship between various theoretically distinct constructs representing EID and related concepts, such as connectedness to nature, the inclusion of nature in the self, and environmental self-identity, is unclear. Recently, Balundé et al. (2019), in a meta-analysis confirmed the strong links (i.e., a great amount of shared variance) between concepts such as identification with the natural environment, (Clayton, 2003) and the emotional aspect of connection with nature (Schultz, 2001). According to the classical test theory (Cronbach and Meehl, 1955; Campbell and Fiske, 1959), this high shared variance could potentially point to these measures being indistinguishable in some cases. In the next paragraph, the issue of the dimensionality of existing scales will be addressed.

### The environmental identity scale

Since theoretical complexity has often emerged in the history of this scale and its structural properties, the following paragraphs will briefly present the psychometric issues that guided the present work.

The EID Scale was developed to measure individual differences in a stable sense of interdependence and connectedness with nature, considering both the cognitive and the emotional facets of people’s relationship with the natural world (Clayton, 2003). The first EID Scale was composed of 24 items with a seven-point Likert scale as an answer format and measured five different aspects of EID: (1) the salience of identity, indicating the degree and significance of an individual’s relationship with nature; (2) the feeling of membership referring to the mode in which nature has a connection to the community with which one identifies oneself; (3) the acceptance of an ideology shared by the group reflected by the endorsement of environmental education and a sustainable lifestyle; (4) positive emotions related to the group identified by the pleasure felt in nature through fulfillment and esthetic enjoyment; (5) an autobiographical component comprising memories of interactions with nature. Although EID is theoretically multidimensional, Clayton (2003) stated that “factor analyses suggest that a single factor accounts for most of the variance” (p. 53), which is why the EID Scale has always been proposed as one-dimensional. However, some studies in the past have proposed different factorial solutions, e.g., Olivos and Aragonés (2011) found four factors plus a one-item factor. Occasionally, a complex structure emerged also out of the borders of Spain, for example, Fritsche and Häfner (2012), employing a set of 12 items issued from the 2003’s EID Scale, found two factors among German students, one interpreted as “Reflecting Contact with Nature” and the other as “Reflecting Self-definition.”

A second, shorter scale was later proposed, which is composed of 11 items (Clayton, 2012; Prévot et al., 2018), for example, “In general, being part of the natural world is an important part of my self-image” and “When I am upset or stressed, I can feel better by spending some time outdoors “communing with nature”. This scale has been used in a study conducted by Scopelliti et al. (2022), which is aimed at gaining a better understanding of the psychosocial determinants of young adults’ pro-environmental behaviors (PEBs), adding the contribution of affect and identity processes to well-established cognitive factors, and the role of intergenerational transmission of ecological values in these mechanisms. Concerning the dimensionality of the scales, most studies are consistent in finding a one-factor solution (e.g., Chew, 2019). Moreira et al. (2021) directly addressed this issue, testing different models through Confirmatory Factor Analysis (CFA), and bifactor exploratory structural equation modeling, finding that EID is an essentially one-dimensional construct.

Literature reports high internal reliability and validity of Clayton’s EID Scale, both in its original 24-item form and in its 11-item version (Clayton et al., 2021).

In 2021 Clayton proposed the Revised EID Scale, an update of the EID scale aiming to have items that are more inclusive of a broad variety of populations and nature experiences, especially to make the scale more applicable to urban and cross-cultural populations (Clayton et al., 2021). For the validation process, the reliability and the validity of the new scale was tested in seven diverse, international samples and reported to be satisfactory. The paper presents the Revised EID scale as composed by one factor, however, in a personal communication, Clayton reported that this solution was chosen for the sake of parsimony and that more factors emerged in preliminary analysis. “In each case the first factor was dominant, explaining from 32% (in the smallest sample, the Chicago students) to 58% (in the MTurk sample) of the total variance. In each sample, all items loaded positively on this first factor. The composition of subsequent factors varied between samples. A factor analysis combining all samples (*N* = 1650) had similar results, with a single factor that explained 48% of variance. A second factor had an eigenvalue just barely over 1.0 (1.05). We concluded that it is most parsimonious to interpret the scale in terms of a single factor” (Clayton, 2022, Personal Communication, March 2).

### An adaptation among Italian pet owners

Even though environmental psychology studies are abundant in Italy, there does not seem to be any available Italian adaptation of the EID Scale, while some translations already exist (see Scopelliti et al., 2022 for the 11-item version). In the framework of an international study on pet owners, involving an Italian sample, it was thus decided to work on an Italian translation and adaptation of the Revised EID Scale.

Even if according to the “pet as ambassador” hypothesis, individuals that owned pets during their childhood are known to have a greater concern for non-pet animals than adults (Paul and Serpell, 1993), studies about EID among pet owners are scarce. More generally, existing evidence shows how early childhood and upbringing experiences can be capital for people’s EID, as well as pro-environmental attitudes (Green et al., 2016; Prévot et al., 2018; Molinario et al., 2020). In this sense, adult pet owners are likely to have a high level of EID and are thus a relevant public to address for studying the scale’s statistical properties.

This report thus intends to present the adaptation of the Revised EID Scale in an Italian sample of pet owners and its preliminary statistical properties. The scale’s structure is going to be explored and compared with the structure proposed for other versions of the same scale.

According to the previous published factorial solutions of the EID, two hypotheses are formulated:

H1: The reliability of the EID Scale is more than 0.70.

H2: The one-factor solution of the EID Scale outperforms the multi-factorial solution.

## Materials and methods

ITC guidelines were followed for developing the adaptation of the scale (Hernández et al., 2020). Most criteria were satisfied with acceptable or excellent criteria, some criteria were not applicable since they refer to psychological tests and some are still to be implemented by future studies (see Section “Discussion”).

More specifically, as a precondition for the adaptation, as explained in the introduction, we evaluated the feasibility and the opportunity of the translation, and we informed the author of the scale (i.e., Susan Clayton) by email about the adaptation project.

As for the development phase, first of all, the study was approved by the faculty’s ethical committee (prot. N. 1214/2021). The adaptation team included three Italian experts in social psychology with professional knowledge of English. Of the three, one was solely involved in the translation process and provided the English-to-Italian translation. The second, who is an expert in environmental psychology, conducted the back translation from Italian to English. The third, which has specific expertise in test construction and validation, was involved with the other two to solve final translation discrepancies and doubts. The team of experts has reviewed, approved, and documented the adequacy and comparability of the instructions, and item content. As in its validated version, the Revised EID Scale was presented as a seven-point Likert scale. A preliminary online pilot study with 88 participants showed that individuals from the target population (i.e., Italian pet owners) do not have difficulties using the proposed procedures.

As for the confirmation step, the scale was included in an online questionnaire on pet attachment. To specifically target pet owners, the questionnaire, hosted on Qualtrics, was distributed through Prolific only to Italian residents owning at least one pet. A question asked participants how many pets they owned (in the present or the past) and how many different types of pets they owned (cats, dogs, fishes, reptiles, small mammals, and birds). Each participant was rewarded with 1.25 £. The sample size was sufficient to carry out exploratory factor analyses. Cronbach’s alpha was employed to test for construct equivalence.

Even though this is not a psychological test expected to have comparable scores and norms among different countries and populations, the present study reports sociodemographic characteristics and their relationships with the adapted scale, aiming to ease comparisons with the samples on which this scale has already been validated.

## Participants

Participants were 163 Italian residents of young age (*M* = 26.61 years, *SD* = 7.05). Male participants were slightly over-represented (90, 55.22%) in comparison to female participants (69, 42.33%), and participants identifying themselves as the third gender were 2.45%. The median completion time of the whole questionnaire was 8.32 min, and participants employing less than 5 min or more than 25 min to complete the whole questionnaire were excluded from the analysis. The sample has been collected within a project on pet owners, so all the participants own one or more pets. Participants reported owning on average nine pets (*SD* = 7.08) of three different types (*SD* = 1.21), such as cats, dogs, and fish.

The sample was very highly educated (47.24% have at least a bachelor’s degree). Six participants report their mother language to be different from Italian. Since answers of non-native Italian speakers were consistent with the other ones for completion time and quality, it was chosen to keep them, in order to account for the wide community of Italian residents whose native language is not Italian.

## Analyses

Analyses were run on RStudio software (version 1.2.1578).

To establish whether the dataset was suitable for exploratory factor analysis (EFA), the sample adequacy, and strength of the intercorrelation of items were examined. The Kaiser–Meyer–Olkin (KMO) measure was used to test sample adequacy. KMO values between 0.7 and 1 indicate adequate sampling. Bartlett’s test of sphericity was used to test the hypothesis that the correlation matrix is an identity matrix, which would indicate that variables are unrelated and therefore unsuitable for structure detection. A parallel analysis and then an EFA (with the minimum residual method and Oblimin rotation) were run to identify the number of factors suggested by the parallel analysis (Tabachnick and Fidell, 2013). A second EFA, set to extract only one factor, was run to test the mono-factorial solution since this was the structure most commonly proposed for this scale. At last, Cronbach’s alpha was used to examine reliability. Cronbach’s alpha higher than 0.70 indicates that the dataset was reliable and acceptable (Nunnally, 1978).

Finally, the present study reports sociodemographic characteristics and their relationship with the adapted scale, aiming to ease comparisons with other samples on which this scale has already been validated. This type of information will enable future systematic reviews and meta-analyses dealing with this scale to compare samples’ characteristics (Shamseer et al., 2015).

## Results

**FIGURE 1:**
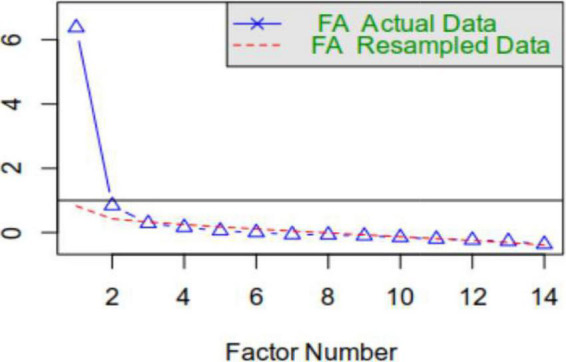
Scree plot of the parallel analysis.

Kaiser–Meyer–Olkin was found to be optimal (*KMO* = 0.93), and Bartlett’s index [*K*^2^(13) = 56.216, *p* < 0.001] was satisfactory. As shown in Figure 1, the parallel analysis (5,000 iterations) suggests the existence of two factors.

As shown in Table 1, the two-factor solution consists of a factor labeled “Connectedness with nature” (nine items) and a factor labeled “Protection of nature” (five items), after examining their content. The two factors are correlated *r* = 0.58 and together explain 52% of the total variance. The one-factor solution, on the other hand, explains 46% of the total variance.

**TABLE 1:** Two-factor and one-factor exploratory factor analysis (EFA) solutions for the Italian revised environmental identity (EID) scale.

			Two-factor solution		One-factor solution
	English item	Italian item	*F1*	*F2*	
1	I like to spend time outdoors in natural settings (such as woods, mountains, rivers, fields, local parks, lake or beach, or a leafy yard or garden).	Mi piace passare il tempo all’aperto in ambienti naturali (come boschi, montagne, fiumi, campi, parchi locali, laghi o spiagge, o un giardino verdeggiante o un orto).	0.86	-0.04	0.77
2	I think of myself as a part of nature, not separate from it.	Penso a me stesso/a come parte della natura, non-separato/a da essa.	0.27	0.49	0.67
3	If I had enough resources such as time or money, I would spend some of them to protect the natural environment.	Se avessi abbastanza risorse, quali tempo e denaro, spenderei parte di esse per proteggere l’ambiente naturale.	0.07	0.66	0.60
4	When I am upset or stressed, I can feel better by spending some time outdoors surrounded by nature.	Quando sono agitato/a o stressato/a, posso sentirmi meglio passando del tempo all’aperto circondato/a dalla natura.	0.83	-0.03	0.75
5	I feel that I have a lot in common with wild animals.	Sento di avere molto in comune con gli animali selvatici.	0.41	0.15	0.51
6	Behaving responsibly toward nature–living a sustainable lifestyle–is important to who I am.	Comportarsi responsabilmente verso la natura–avere uno stile di vita sostenibile–è importante per ciò che sono io.	-0.11	0.86	0.58
7	Learning about the natural world should be part of everyone’s upbringing.	Conoscere il mondo naturale dovrebbe essere parte dell’educazione di tutti.	0.17	0.57	0.63
8	If I could choose, I would prefer to live where I can have a view of the natural environment, such as trees or fields.	Se potessi scegliere, preferirei vivere dove posso avere un affaccio sull’ambiente naturale, come alberi o campi.	0.37	0.31	0.61
9	An important part of my life would be missing if I was not able to get outside and enjoy nature from time to time.	Mi mancherebbe una parte importante della mia vita, se non fossi in grado di uscire e godermi la natura ogni tanto.	0.78	0.07	0.80
10	I think elements of the natural world are more beautiful than any work of art.	Penso che gli elementi del mondo naturale siano più belli di qualsiasi opera d’arte.	0.40	0.12	0.49
11	I feel refreshed when I spend time in nature.	Mi sento riposato/a quando spendo tempo nella natura.	0.96	-0.06	0.84
12	I consider myself a steward of our natural resources.	Mi considero un/a custode delle nostre risorse naturali.	0.04	0.69	0.60
13	I feel comfortable out in nature.	Mi sento a mio agio fuori nella natura.	0.73	0.13	0.80
14	I enjoy encountering elements of nature, like trees or grass, even when I am in a city setting.	Mi piace quando mi imbatto in elementi della natura, come alberi o erba, anche quando sono in un contesto urbano.	0.44	0.31	0.68
	Cronbach’s α		0.89	0.83	0.92

*F1*: “Connectedness with nature”; *F2*: “Protection of nature.”

It should be noted that, in the two-factor solution, item 8 has a low loading (only slightly higher than the 0.32 threshold) (Hair et al., 2006; Tabachnick and Fidell, 2013) on both factors. However, if, consistently with its content, it is included in the “Connectedness with nature” factor, it does not seem to negatively affect its Cronbach’s alpha. Moreover, removing it does not change the overall factors’ structure.

Cronbach’s alpha has satisfactory values both for the one-factor and the two-factor solutions, confirming H1.

The only way to compare EFA solutions is by comparing explained variance percentages. Since the two-factor solution explains a higher percentage of variance, it seems like a better solution. The two-factor solution was also the one suggested by the parallel analysis as well as by eigenvalues (the first two factors were the only ones with an eigenvalue above 1, i.e., 6.87 and 1.40). Thus, H2 was disconfirmed.

Here, the descriptive characteristics of the two-factor solution and the correlations between the factors and the sociodemographic variables are presented.

As shown in Table 2, the distribution of both factors was moderately skewed with participants giving in general high-value answers.

**TABLE 2:** Descriptive statistics of the two factors of the scale.

	Mean	SD	Median	Min	Max	Skew	Kurtosis	SE
Connectedness with nature	5.39	1.01	5.44	1.67	7.00	−0.76	0.55	0.08
Protection of nature	5.55	0.96	5.60	2.20	7.00	−0.68	0.22	0.08

As shown in Table 3, no differences between age and education were found. Descriptive results were also independent of the number and variety of pets owned. Student’s *t*-tests showed no differences between men and women in both factors.

**TABLE 3:** Correlations between the two environmental identity (EID) scale factors, age, education, number of pet types, and number of pets (Spearman’s ρ).

		1	2	3	4	5	6
1	Connectedness with nature	1					
2	Protection of nature	0.73***	1				
3	Age	−0.08	0.03	1			
4	Education	0.03	0.01	0.40***	1		
5	Number of pet kinds	0.01	0.00	−0.08	−0.11	1	
6	Number of pets	0.00	−0.03	−0.04	−0.18*	0.74***	1

*p < 0.05; ***p < 0.001.

## Discussion

The adaptation procedure allowed the production of a reliable and acceptable version of the Revised EID Scale. Moreover, the EFA provided an adequate, albeit preliminary factor solution. The solution has good internal reliability (confirming H1) but is different from the only study currently presenting factor analyses about this scale (i.e., Clayton et al., 2021). Here, the scale’s structure is better described by a two-factor solution, disconfirming H2. Since the original validation study presents only a CFA and no EFA, it is impossible to know if a bi-factor solution would have been appropriate in that case.

However, the two factors proposed in the present study are consistent with other solutions that emerged for the previous versions of the EID Scale, especially with the four-factor solution proposed by Olivos and Aragonés (2011; see also Larionov, 2020). Even though that solution had more factors than the present one, it can be noted how the “Connectedness to nature” factor emerging here is close to the factors “Enjoying Nature” and “Appreciation of Nature,” while the “Protection of nature” factor is linked to the “Environmentalism” factor, and also, partially to the “Environmental Identity” factor emerged in Spain.

The skewed distribution of both factors is possibly determined by the present sample consisting of pet owners since they are known to have stronger environmental concerns than the average (Auger and Amiot, 2017). At the same time, Clayton already reported that in the original samples “all item means were above the neutral midpoint” (Clayton et al., 2021 p. 8), so the issue might not only be related to the specificities of this sample.

Similar to the cross-cultural validation (2021), no relevant associations between gender, age, and/or education, and EID emerged in this study on Italian pet owners, suggesting that EID might be independent of the main sociodemographic variables also in this specific sample. Moreover, this study shows that the number and the variety of pets do not seem to affect the EID scores. At first sight, this result seems to be inconsistent with the “pet as ambassador” hypothesis (Serpell and Paul, 1994); however, it should be considered that all the participants in this study are pet owners. In this sense, it is possible that including non-pet owners would show that owning even just one pet is associated with a higher EID. Thus, it might not be a matter of how many pets one has but simply being or not being a pet owner might make a difference. This hints that future studies should consider populations with different characteristics (e.g., lack of pets, specific marital status, and presence of children), as well as representative samples of the general population. It should be noted that the ITC guidelines suggest following some extra steps that are not presented in this preliminary report (Hernández et al., 2020). Other validation tests should be run, especially for what concerns discriminant validity, convergent validity, and test–retest validity. For better comparisons with other adaptations of this scale, construct equivalence, method equivalence, and measurement equivalence (item functioning) should be tested more thoroughly.

More generally, results should be considered with caution, and the structure should be tested again on a bigger and more representative sample of the Italian population. Moreover, future studies should test this structure with more and more complex analyses such as CFA and exploratory structural equation modeling that were not run here because of the relatively small sample size.

## Data availability statement

The raw data supporting the conclusions of this article will be made available by the authors, without undue reservation.

## Ethics statement

The studies involving human participants were reviewed and approved by Department of Psychology of Developmental and Socialization Processes, Sapienza University of Rome–prot. N. 1214/2021. The participants provided their written informed consent to participate in this study.

## Author contributions

SA was responsible for conception, design of the study, and the organization of the database. SA and OM performed the statistical analysis and wrote the manuscript together. Both authors contributed to the article and approved the submitted version.
